# Multisystem Inflammatory Syndrome after Breakthrough SARS-CoV-2 Infection in 2 Immunized Adolescents, United States

**DOI:** 10.3201/eid2807.220560

**Published:** 2022-07

**Authors:** Lyndsey D. Cole, Molly Slate, Samantha Minneman, Michael J. Bozzella

**Affiliations:** University of Colorado, Aurora, Colorado, USA

**Keywords:** COVID-19, multisystem inflammatory syndrome in children, MIS-C, SARS-CoV-2, viruses, vaccine-preventable diseases, inflammation, critical illnesses, adolescents, respiratory infections, zoonoses, coronavirus disease, severe acute respiratory syndrome coronavirus 2, United States

## Abstract

Eight weeks after having laboratory-confirmed SARS-CoV-2 breakthrough infections, 2 otherwise healthy, fully immunized adolescent patients in the United States who were experiencing related signs and symptoms were diagnosed with multisystem inflammatory syndrome in children. Our findings indicate that COVID-19 vaccination does not completely protect adolescents against multisystem inflammatory syndrome.

Multisystem inflammatory syndrome in children (MIS-C) is a hyperinflammatory illness occurring after SARS-CoV-2 infection; ≈30% of case-patients are adolescents (*1*). The United States reported 7,880 MIS-C cases and 66 deaths as of March 28, 2022 (*1*). MIS-C is an exception to the lower incidence and death from SARS-CoV-2–associated health conditions in children than adults (*2*). In April–June 2020, estimated MIS-C incidence across 7 US jurisdictions was 316 (95% CI 278–357) cases per 1 million SARS-CoV-2 infections in persons <21 years of age (*3*). The small but serious risk of MIS-C has been cited in support of pediatric SARS-CoV-2 immunization (*4*,*5*).

The BNT162b2 Pfizer-BioNTech mRNA COVID-19 vaccine (https://www.pfizer.com) was approved in December 2020 for children ≥16 years of age and in May 2021 for children 12–15 years of age (*6*). As of April 20, 2022, a total of 68% of US children 12–17 years of age had been fully immunized (*6*). Data about the effects of SARS-CoV-2 immunization on MIS-C are limited, although some evidence suggests the vaccine offers protection against MIS-C in adolescents (*7*,*8*). We describe 2 cases of MIS-C after breakthrough SARS-CoV-2 infection in fully immunized adolescents. 

## The Study

In the first case, headache and myalgia developed in a healthy 14-year-old boy (day 1 of illness); by day 7, fever, abdominal pain, diarrhea, emesis, bloodshot eyes, red cracked lips, and rash had also developed. On day 10, he was brought for treatment to the emergency department and admitted to a quaternary-care pediatric hospital.

Three months earlier, he completed the Pfizer-BioNTech 2-dose COVID-19 vaccine series (Figure). One month later, he experienced 3 days of coughing and congestion and tested positive by PCR for SARS-CoV-2 infection, from which he recovered. 

**Figure:**
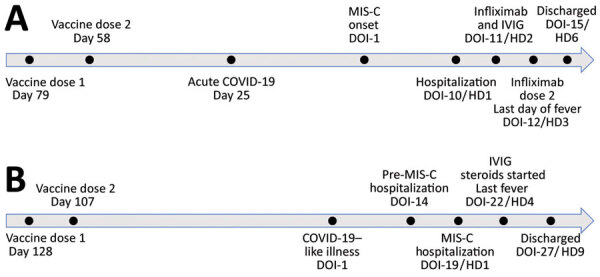
Time courses for vaccination, illness, diagnosis, and treatment for 2 adolescent MIS-C case-patients, United States. A) Case-patient 1; B) case-patient 2. DOI, day of illness (since onset); HD, hospital day; IVIG, intravenous immunoglobulin; MIS-C, multisystem inflammatory syndrome in children.

At hospital admission, examination noted sickly appearance, fever (39.1°C), tachycardia, rash, conjunctivitis, cracked lips, and abdominal tenderness. Laboratory testing revealed hyponatremia; thrombocytopenia; lymphopenia; and elevated C-reactive protein (CRP), N-terminal pro-brain natriuretic peptide (NT-proBNP), and liver function test levels (Table 1). Echocardiogram revealed trivial pericardial effusion. Abdominal ultrasound and chest radiograph results were unremarkable. SARS-CoV-2 spike and nucleocapsid IgG results were positive. Other infectious condition test results were negative (Table 2).

**Table 1:** Laboratory results for 2 adolescent MIS-C case-patients, by day in hospital, United States*

Result†	Case-patient 1, hospitalization for MIS-C							Case-patient 2‡							
								Pre–MIS-C hospitalization			Hospitalization for MIS-C				
	HD1	HD2	HD3	HD4	HD5	HD6		HD1	HD4		HD1	HD3	HD5	HD7	HD9
Leukocytes, × 10^9^ cells/L	↓3.6	↓4.1	↓2.3	↓2.6	↓3.1	↓2.7		11.9	NA		7.8	5.8	↓2.9	5.4	9.0
Hemoglobin, g/dL	12.5	13.1	12.4	12.2	12.4	↓10.5		13.5	NA		13.8	↓10.8	↓10.4	↓10.3	↓10.5
Platelets, 10^9^/L	↓98	↓109	188	237	321	362		290	NA		373	265	NA	305	331
Absolute neutrophil count, × 10^9^ cells/L	2.4	3.3	↓1	↓0.9	↓1	↓0.6		NA	NA		↓1.4	1.8	2.0	3.6	4.7
Absolute lymphocyte count, × 10^9^ cells/L	1	↓0.6	1.1	1.3	1.7	1.8		NA	NA		5.8	2.3	↓0.6	1.2	2.8
ESR, mm/h	NA	6	7	↑23	↑24	↑25		↑18	NA		↑22	↑96	NA	NA	NA
Sodium, mmol/L	↓130	↓136	↓134	↓133	↓132	137		↓135	NA		↓136	137	↓127	↓136	139
Creatinine, mg/dL	↑1.2	↑1.0	↑0.9	0.7	↑0.8	0.6		0.7	NA		↑1.0	↑2.2	↑3.9	↑2.3	↑1.3
AST, U/L	↑165	↑207	↑131	↑114	↑200	↑164		↑297	↑164		↑102	↑169	↑53	28	33
ALT, U/L	↑221	↑243	↑196	↑167	↑195	↑162		↑249	↑269		↑188	↑177	↑123	↑57	45
GGT, U/L	↑126	↑137	↑129	↑128	↑136	NA		NA	NA		↑342	↑209	NA	NA	NA
LDH, U/L	↑1,484	NA	↑1,155	↑928	↑863	NA		↑1,313	NA		↑982	NA	NA	NA	NA
C-reactive protein, mg/L	↑135	↑86	↑64	↑36	↑19	↑11		↑23	NA		↑17	↑47	↑56	↑19	10
Ferritin, ng/mL	↑750	NA	↑576	↑626	↑754	↑578		↑593	NA		191	284	NA	NA	NA
Albumin, g/dL	NA	↓2.9	↓2.8	↓2.8	↓3.1	↓3.1		↓3.1	↓3.2		3.6	↓2.6	3.0	↓3.1	↓3.1
aPTT, s	↑42.9	36.5	↑38.9	↑39.4	NA	NA		↓20.5	NA		28.9	31.3	NA	NA	NA
PT, s	14	12.6	13.5	13.7	14.1	13.5		13.4	NA		13.5	14.7	NA	NA	NA
Fibrinogen, mg/dL	328	354	302	269	317	NA		258	NA		413	NA	NA	NA	NA
D-dimer, μg/mL	NA	↑>4	↑>4	↑>4	↑3.1	NA		NA	NA		↑2.8	NA	NA	NA	NA
Troponin-I, ng/mL	0.03	↑0.04	↑0.05	0.03	0.02	NA		<0.02	NA		<0.02	NA	NA	NA	NA
NT-proBNP, pg/mL	↑365	↑477	↑601	↑1,020	↑212	NA		66	NA		85	↑3,190	↑3,360	↑1,300	↑1,590

*ALT, alanine aminotransferase; aPTT, activated partial thromboplastin time; AST, aspartate aminotransferase; ESR, erythrocyte sedimentation rate; GGT, gamma-glutamyl transpeptidase; HD, hospital day; LDH, lactate dehydrogenase; MIS-C, multisystem inflammatory syndrome in children; NT-proBNP, N-type pro-brain natriuretic peptide; PT, prothrombin time; ↑, increased outside of reference range; ↓, decreased outside of reference range. †Reference ranges: leukocytes, 5.2–9.7 × 10^9^ cells/L; hemoglobin, 11.8–15.8 g/dL; platelets, 150–500 × 10^9^/L; absolute neutrophil count, 1.8–6.6 × 10^9^ cells/L; absolute lymphocyte count, 1.0–4.8 × 10^9^/L; ESR, <15 mm/h; sodium, 137–145 mmol/L; creatinine, 0.2–0.7 mg/dL; AST, 15–40 U/L; ALT, <50 U/L; GGT, 10–28 U/L; LDH, 360–730 U/L; C-reactive protein, 0–10 mg/L; ferritin, 6–464 ng/mL; albumin 3.5–5 g/dL; aPTT, 23.5–37.5 s; PT, 11.5–15.0 s; fibrinogen, 200–450 mg/dL; D-dimer, <0.5 μg/mL; troponin-I, <0.03 ng/mL; NT-proBNP, <125 pg/mL ‡Omitted data for case-patient 2 from HD2 and HD3 of pre–MIS-C hospitalization and data from HD4, HD6, and HD8 of hospitalization for MIS-C were unnecessary for demonstrating clinical trends

**Table 2:** Infectious laboratory results for 2 adolescents after multisystem inflammatory syndrome in children symptom onset and throughout hospitalization, United States*

Laboratory test	Case-patient 1	Case-patient 2
SARS-CoV-2 spike IgG	Positive	NA
SARS-CoV-2 nucleocapsid IgG	Positive	Positive
SARS-CoV-2 PCR	Negative	Positive†
Other respiratory pathogen panel PCR	Negative	Negative
Blood culture, peripheral, 2 sets	Negative	Negative
Urine culture	Negative	Negative
Epstein Barr virus ab panel	Consistent with prior infection	Negative
Gastrointestinal pathogen panel PCR	Negative	NA
Group A *Streptococcus* throat PCR	Negative	NA
Quantiferon tuberculosis gold	Negative	NA
HIV Ag/Ab, 4th-generation	NA	Nonreactive
Rapid plasma regain	NA	Nonreactive
Parvovirus IgM and IgG	NA	Negative
Anti-streptolysin-O	NA	Negative
Anti-deoxyribonuclease B	NA	Negative

*Ag, antigen; Ab, antibody. †Case-patient 2 had a positive SARS-CoV-2 PCR result on day-of-illness 11, followed by a negative result on day-of-illness 19.

On the patient’s first day of hospitalization, the infectious diseases section was consulted, and we determined that the patient’s illness met Centers for Disease Control and Prevention MIS-C criteria (*1*). The next day, he received infliximab (10 mg/kg), followed by intravenous immunoglobulin. Rash, headache, conjunctivitis, and CRP improved; however, fever, malaise, and nausea persisted, and cardiac markers rose, prompting a second infliximab dose on hospitalization day 3. His fever subsided, and signs, symptoms, and laboratory results improved (Table 1). On hospitalization day 5, echocardiogram showed no effusion; he was discharged the next day.

Infectious diseases follow-up 3 weeks after hospital discharge revealed fatigue and occasional mild abdominal pain but normalized laboratory results. Cardiology follow-up 6 weeks after hospital discharge revealed ongoing fatigue. An echocardiogram showed new mild left main coronary artery enlargement (Z-score +2.7). We planned no interventions beyond interval outpatient monitoring.

In the second case, fever and fatigue, followed by congestion, cough, myalgias, headache, nausea, and vomiting, developed in an otherwise healthy 14-year-old girl (day 1 of illness). On day 3, rapid SARS-CoV-2 and influenza test results were negative. On day 12, she was brought to the emergency department for persistent fever, headache, cough, and vomiting. Three months before her illness, she had completed the 2-dose Pfizer-BioNTech COVID-19 vaccine series (Figure).

Results from computed tomography of her head and chest radiograph were unremarkable. SARS-CoV-2 PCR was positive. She was prescribed amoxicillin for possible sinusitis and discharged. On day 14, she returned to the hospital for dyspnea and required low-flow oxygen for hypoxemia. Electrocardiogram, troponin, and NT-proBNP test results were normal. She was admitted and received 1 dose of remdesivir, which we discontinued because of elevated liver function test results (Table 1), and 2 doses of dexamethasone; we also discontinued amoxicillin. She improved and was discharged on day 18. However, she returned the next day with recrudescent fever, emesis, and new diffuse rash, including on her palms and soles. Laboratory testing demonstrated elevated CRP, D-dimer, liver function, NT-proBNP, and creatinine levels (Table 1). Abdominal ultrasound and computed tomography showed incidentally enlarged kidneys. We empirically started clindamycin and ceftriaxone treatment and readmitted her.

At readmission on day 19, differential diagnoses included MIS-C, acute COVID-19 with hyperinflammation, sepsis, toxic shock syndrome, drug reaction, and vasculitis or other autoimmune disease. Echocardiogram results were unremarkable. A SARS-CoV-2 nucleocapsid IgG test was positive. Additional infectious and rheumatologic test results were negative (Table 2). After discussion among multidisciplinary specialists, we considered MIS-C most likely, with a suspicion that her earlier symptoms might have resulted from acute COVID-19, which evolved over 3 weeks into MIS-C. We discontinued antibiotics.

On day 4 of her second hospitalization, she received intravenous immunoglobulin and methylprednisolone. Her fever quickly subsided, signs and symptoms resolved, and laboratory results improved (Table 1). On hospitalization day 9, she was discharged with an oral prednisone taper. Infectious disease follow-up 3 weeks after discharge revealed mild fatigue and headaches but normalized laboratory results. Cardiology follow-up through 12 weeks after discharge indicated fatigue, but echocardiogram and exercise stress testing results were unremarkable.

## Conclusions

We report 2 cases in which fully vaccinated, otherwise healthy adolescent patients were diagnosed with MIS-C after laboratory-confirmed SARS-CoV-2 breakthrough infections. Diagnoses were based on CDC criteria, and clinical findings were similar to descriptions from cohort studies published elsewhere (*9*–*11*). Based on Brighton Collaboration MIS-C case definitions (https://brightoncollaboration.us), we considered MIS-C diagnosis definitive in case-patient 1 and probable in case-patient 2, although her condition had some features more suggestive of acute COVID-19 with hyperinflammation (*12*). Neither case met SARS-CoV-2 vaccine-associated MIS-C criteria (*12*).

As adolescent SARS-CoV-2 immunization rates have increased, so has interest in the effects of vaccination on adolescent MIS-C. In a fall 2021 study in France, 0/33 adolescents with MIS-C were fully vaccinated against COVID-19 (*7*). Seven (21%) had received 1 shot of a 2-dose vaccine; hazard ratio for MIS-C was 0.09 (95% CI 0.04–0.21) among the partially vaccinated compared with unvaccinated patients (p<0.001) (*7*). A January 2022 report estimated 91% (95% CI 78%–97%) vaccine effectiveness against MIS-C based on 102 case-patients and 181 hospitalized controls at 24 US pediatric hospitals during July–December 2021 (*8*). Only 5% of MIS-C case-patients were fully vaccinated, compared with 36% of controls. None of the 5 fully COVID-19–vaccinated MIS-C patients, compared with 39% of unvaccinated patients, required invasive mechanical ventilation, vasoactive medications, or extracorporeal membrane oxygenation (*8*), suggesting decreased MIS-C severity in vaccinated patients, similar to our 2 case-patients. Another US review reported mechanical ventilation use in 14% and vasopressor use in 38% of 21 patients with MIS-C after SARS-CoV-2 vaccination; 52% had received only 1 of 2 vaccine doses, and median time from second dose to MIS-C onset was 5 days, shorter than expected to reach full vaccine efficacy (*13*). Although those findings could suggest vaccine-associated MIS, 71% of patients showed evidence of SARS-CoV-2 breakthrough infection (*13*). Our case-patients demonstrated clear breakthrough infections, ruling out vaccine-associated MIS based on Brighton criteria (*12*).

Although COVID-19 vaccines appear to be effective against MIS-C (*7*,*8*), whether effectiveness results from decreased risk of acquiring SARS-CoV-2 or reduced likelihood of developing MIS-C after a breakthrough infection remains unclear. Because vaccine effectiveness against older versus newer variants might differ, potential vaccine effect on MIS-C rates after breakthrough infections becomes more important (*14*). Additional research is needed, particularly among age groups younger than those in our study, which account for most MIS-C cases. However, our findings indicate that even full COVID-19 vaccination in adolescents is not 100% effective against MIS-C.

## References

[1] Centers for Disease Control and Prevention. Health department-reported cases of multisystem inflammatory syndrome in children (MIS-C) in the United States. 2021 [cited 2022 Apr 28]. https://www.cdc.gov/mis-c/cases/index.html

[2] Mehta NS, Mytton OT, Mullins EWS, Fowler TA, Falconer CL, Murphy OB, et al. SARS-CoV-2 (COVID-19): what do we know about children? A systematic review. Clin Infect Dis. 2020;71:2469–79. 10.1093/cid/ciaa556 32392337 PMC7239259

[3] Payne AB, Gilani Z, Godfred-Cato S, Belay ED, Feldstein LR, Patel MM, et al.; MIS-C Incidence Authorship Group. Incidence of multisystem inflammatory syndrome in children among US persons infected with SARS-CoV-2. JAMA Netw Open. 2021;4:e2116420. 10.1001/jamanetworkopen.2021.16420 34110391 PMC8193431

[4] Klass P, Ratner AJ. Vaccinating children against Covid-19—the lessons of measles. N Engl J Med. 2021;384:589–91. 10.1056/NEJMp2034765 33471977

[5] Castagnola E, Mariani M, Sticchi C, Sticchi L, Spiazzi R, Caorsi R, et al. Incidence rate of MIS-C in paediatrics: A good reason to vaccinate children against SARS-CoV-2. Acta Paediatr. 2022;111:123–4. 10.1111/apa.16081 34432905 PMC8653129

[6] American Academy of Pediatrics. Children and COVID-19 vaccination trends. Summary of data publicly reported by the Centers for Disease Control and Prevention [cited 2022 Apr 28]. https://www.aap.org/en/pages/2019-novel-coronavirus-covid-19-infections/children-and-covid-19-vaccination-trends

[7] Levy M, Recher M, Hubert H, Javouhey E, Fléchelles O, Leteurtre S, et al. Multisystem inflammatory syndrome in children by COVID-19 vaccination status of adolescents in France. JAMA. 2022;327:281–3. 10.1001/jama.2021.23262 34928295 PMC8689418

[8] Zambrano LD, Newhams MM, Olson SM, Halasa NB, Price AM, Boom JA, et al.; Overcoming COVID-19 Investigators. Effectiveness of BNT162b2 (Pfizer-BioNTech) mRNA vaccination against multisystem inflammatory syndrome in children among persons aged 12–18 years—United States, July–December 2021. MMWR Morb Mortal Wkly Rep. 2022;71:52–8. 10.15585/mmwr.mm7102e1 35025852 PMC8757620

[9] Centers for Disease Control and Prevention. Multisystem inflammatory syndrome in children (MIS-C) associated with coronavirus disease 2019 (COVID-19). 2020 [cited 2022 Jan 9]. https://emergency.cdc.gov/han/2020/han00432.asp

[10] Feldstein LR, Tenforde MW, Friedman KG, Newhams M, Rose EB, Dapul H, et al.; Overcoming COVID-19 Investigators. Characteristics and outcomes of US children and adolescents with multisystem inflammatory syndrome in children (MIS-C) compared with severe acute COVID-19. JAMA. 2021;325:1074–87. 10.1001/jama.2021.2091 33625505 PMC7905703

[11] Godfred-Cato S, Bryant B, Leung J, Oster ME, Conklin L, Abrams J, et al.; California MIS-C Response Team. COVID-19–associated multisystem inflammatory syndrome in children—United States, March–July 2020. MMWR Morb Mortal Wkly Rep. 2020;69:1074–80. 10.15585/mmwr.mm6932e2 32790663 PMC7440126

[12] Vogel TP, Top KA, Karatzios C, Hilmers DC, Tapia LI, Moceri P, et al. Multisystem inflammatory syndrome in children and adults (MIS-C/A): Case definition & guidelines for data collection, analysis, and presentation of immunization safety data. Vaccine. 2021;39:3037–49. 10.1016/j.vaccine.2021.01.054 33640145 PMC7904456

[13] Yousaf AR, Cortese MM, Taylor AW, Broder KR, Oster ME, Wong JM, et al.; MIS-C Investigation Authorship Group. Reported cases of multisystem inflammatory syndrome in children aged 12-20 years in the USA who received a COVID-19 vaccine, December, 2020, through August, 2021: a surveillance investigation. Lancet Child Adolesc Health. 2022;6:303–12. 10.1016/S2352-4642(22)00028-1 35216660 PMC8864018

[14] Cele S, Jackson L, Khoury DS, Khan K, Moyo-Gwete T, Tegally H, et al.; NGS-SA; COMMIT-KZN Team. Omicron extensively but incompletely escapes Pfizer BNT162b2 neutralization. Nature. 2022;602:654–6. 10.1038/s41586-021-04387-1 35016196 PMC8866126

